# Progress in Preventing Perinatal HIV Transmission in the United States^1^

**DOI:** 10.3201/eid1011.040622_01

**Published:** 2004-11

**Authors:** Mary Glenn Fowler, Patricia Garcia, Celine Hanson, Stephanie Sansom

**Affiliations:** * Centers for Disease Control and Prevention, Atlanta, Georgia, USA;; † Northwestern University, Chicago, Illinois, USA;; ‡ Baylor Medical Center, Houston, Texas, USA

**Keywords:** HIV, perinatal transmission, conference summary

During the past 10 years, perinatal HIV transmission has decreased dramatically in the United States; the reduction is directly related to use of antiretroviral and obstetric interventions. However, some infants continue to become infected with HIV, which reflects missed opportunities for prevention.

## Mother-to-Child HIV Transmission

As the HIV epidemic in the United States continues to evolve, increasing numbers of adolescent girls and women are affected. The proportion of overall AIDS cases reported among women and adolescent girls has risen from 8% in 1986 to 26% in 2001. Heterosexual transmission is now the most common risk factor for acquiring HIV among women and adolescent females. Black and Hispanic women are disproportionately represented among AIDS cases in women in the United States.

The demographics of the pediatric HIV epidemic in the United States closely mirror those of the epidemic in women. Approximately 10,000 perinatally infected children and adolescents are living with HIV; with effective treatments, most are approaching or have entered adolescence. The number of new perinatal infections each year has steadily declined since 1994, when a zidovudine regimen given prenatally, intrapartum, and to the newborn was shown to reduce the risk of mother-to-child transmission by two thirds. Transmission risk declined from 20% to 25% before any interventions to <2% with use of combination antiretroviral drugs during pregnancy and obstetrical interventions, such as scheduled cesarean section before labor onset. However, the Centers for Disease Control and Prevention (CDC) estimates that since 2000, 280–370 infants continue to become perinatally infected each year. Risk factors associated with transmission include lack of receipt of prenatal antiretroviral drugs, maternal clinical status, detectable maternal viral load at delivery, low maternal CD4 count, and immunogenetic host factors.

## Prenatal HIV Testing

Since the results of the clinical trial using zidovudine were announced in 1994, CDC has worked closely with its federal, state, and community partners to promote prenatal HIV testing for all pregnant women. Effective interventions can then be offered to women identified as HIV-infected, both for their own treatment and for perinatal prevention. Beginning in 1999, CDC funds have been made available to high prevalence states for perinatal HIV prevention programs and enhanced perinatal surveillance and to national organizations to develop perinatal HIV prevention training and education materials for healthcare providers and patients. Current U.S. public efforts include promotion of universal prenatal HIV screening for all pregnant women by including HIV testing as part of the standard battery of prenatal tests unless the woman declines testing (the "opt-out" approach to voluntary prenatal screening). For those women whose HIV status is still unknown at delivery, routine rapid HIV testing is being promoted.

## Incorporating Rapid HIV Testing at Labor and Delivery

Enhanced perinatal surveillance data from CDC suggest that the HIV status is known for >90% of women before delivery. However, 40% of infected infants in recent years have been born to the subset of women whose HIV status was still unknown to their provider at the time of labor. Recent developments, including the licensing of several rapid HIV tests, have made rapid HIV testing possible for women in labor. Antiretroviral interventions can then be provided for perinatal HIV prevention to those women with positive results and to their newborns. The MIRIAD (Mothers and Infants Rapid Intervention at Delivery) research study conducted at 16 high-prevalence hospitals in the United States demonstrated the feasibility of obtaining informed consent and offering rapid testing to women in labor. On the basis of findings from the MIRIAD study and the availability of recently licensed rapid testing kits, CDC recommends and promotes routine rapid HIV testing using the opt-out approach for all women in labor whose HIV status is still unknown.

